# Biofilm-Associated Candidiasis: Pathogenesis, Prevalence, Challenges and Therapeutic Options

**DOI:** 10.3390/ph18040460

**Published:** 2025-03-25

**Authors:** Valerie Amann, Ann-Kathrin Kissmann, Carolina Firacative, Frank Rosenau

**Affiliations:** 1 Institute of Pharmaceutical Biotechnology, Ulm University, 89081 Ulm, Germany; valerie.amann@uni-ulm.de (V.A.); ann-kathrin.kissmann@uni-ulm.de (A.-K.K.); 2 Studies in Translational Microbiology and Emerging Diseases (MICROS) Research Group, School of Medicine and Health Sciences, Universidad del Rosario, Bogota 111221, Colombia; cfiracative@gmail.com

**Keywords:** *Candida*, candidiasis, *C. albicans*, *C. parapsilosis*, *C. auris*, biofilm, resistance, fungicides, anti-biofilm, antimicrobial peptides

## Abstract

The rising prevalence of fungal infections, especially those caused by *Candida* species, presents a major risk to global health. With approximately 1.5 million deaths annually, the urgency for effective treatment options has never been greater. *Candida* spp. are the leading cause of invasive infections, significantly impacting immunocompromised patients and those in healthcare settings. *C. albicans*, *C. parapsilosis* and the emerging species *C. auris* are categorized as highly dangerous species because of their pathogenic potential and increasing drug resistance. This review comparatively describes the formation of microbial biofilms of both bacterial and fungal origin, including major pathogens, thereby creating a novel focus. Biofilms can further complicate treatment, as these structures provide enhanced resistance to antifungal therapies. Traditional antifungal agents, including polyenes, azoles and echinocandins, have shown effectiveness, yet resistance development continues to rise, necessitating the exploration of novel therapeutic approaches. Antimicrobial peptides (AMPs) such as the anti-biofilm peptides Pom-1 and Cm-p5 originally isolated from snails represent promising candidates due to their unique mechanisms of action and neglectable cytotoxicity. This review article discusses the challenges posed by *Candida* infections, the characteristics of important species, the role of biofilms in virulence and the potential of new therapeutic options like AMPs.

## 1. *Candida* and Candidiasis

The incidence of invasive fungal infections in humans has reached alarming levels. Worldwide, billions of people are infected with these eukaryotic pathogens and about 1.5 million patients die every year. Different species of the genera *Candida*, *Aspergillus* and *Cryptococcus* are responsible for 90% of cases of fungal infections with high mortality rates [1]. In fact, four species of these pathogens are classified by the World Health Organization (WHO) in the critical priority group [2]. Among these three highly dangerous microorganisms, *Candida* spp. is characterized by its high pathogenicity, which has been identified as the most common cause of invasive infections. As a result of this and the dramatic increase in infections associated with drug-resistant *Candida* spp., the US Centers for Disease Control and Prevention have identified this pathogen as a major risk to public health [1].

*Candida* spp. occurs naturally on the skin, in the gastrointestinal (GI) tract and on mucosal surfaces, including the oral and vaginal cavities of up to 70% of healthy individuals [3,4]. Under certain conditions, however, this fungus can overgrow and change its lifestyle into a pathogenic type, leading to primary infections in those body areas in which it naturally inhabits [5]. Risk factors for the development of a so-called “candidiasis” are prolonged stay in the intensive care unit (ICU), use of prosthetic material (e.g., central venous catheters), surgery on the GI tract (increased permeability of the intestinal epithelium can facilitate the translocation of the organism from the intestine to the bloodstream), polytrauma, advanced age, immunosuppression, neutropenia, solid tumors and hematological malignancies, as well as various drug treatments with agents such as antibiotics or corticosteroids [1,6,7,8,9,10,11,12,13,14,15]. The spectrum of resulting diseases ranges from superficial infections of the mucous membranes, such as oropharyngeal or vulvovaginal candidiasis, to deep-seated, life-threatening diseases caused by dissemination, such as invasive candidiasis (secondary infections affecting organs like the heart, lungs, bones and brain) [16,17].

As one of the most widespread fungal infections, candidiasis affects approximately 250,000 to 700,000 individuals worldwide each year, with an incidence rate of 2 to 14 cases per 100,000 people. The mortality rate is between 40% and 55%, and about 79 cases are diagnosed daily [17,18,19]. New risk groups emerged during the COVID-19 pandemic, which potentially increased the incidence rate mentioned above [20]. In the United States, *Candida* infections are the fourth most common hospital-related bloodstream infection [21], with numerous cases in certain clinical areas such as the ICU (60%) as well as in cancer and transplant facilities (13%) [22,23]. The number of *Candida* spp. infections outside of hospital settings is even higher. For example, between 50 and 70% of women in their childbearing years will experience vulvovaginal candidiasis at least once, and 5 to 8% will suffer from recurrent infections [24].

These serious diseases are triggered by around 15 species of *Candida*, which are part of the roughly 200 species that have been documented so far [8,24]. Among these, the six organisms *Candida albicans*, *Candida parapsilosis*, *Nakaseomyces glabratus* (formerly *Candida glabrata*), *Candida tropicalis*, *Pichia kudriavzevii* (formerly *Candida krusei*) and, in some regions of the world already, *Candida auris* are the most common pathogens causing about 95% of invasive disease [25,26,27,28,29,30]. *C. albicans* and *C. parapsilosis*, particularly, are well adapted to various host environments due to their frequent coexistence with different microbiota members and their genetic, morphological and biochemical flexibility, significantly influencing the course and outcome of disease. Certain *Candida* species are linked to specific risk groups, suggesting that differences in their colonization and survival strategies only result in infections under particular conditions. For example, *C. parapsilosis* is more often linked to infections in neonates than in adults and is a common pathogen in catheter-related infections [31].

### 1.1. C. albicans

The species most frequently isolated and known for its virulence is *C. albicans* [21]. With its remarkable ability to grow both as round budding yeast cells and as pseudo or true hyphae [32,33,34], this polymorphic yeast is a natural member of the human microbiome, colonizing areas such as the oropharynx, genitals, and gastrointestinal mucosa in healthy individuals [35,36,37]. The genetic instability of the naturally diploid genome of *C. albicans* leads to this phenotypic diversity, making it one of the key factors contributing to its virulence [38]. This pathogen’s unique features allow it to flourish in host niches with changing environmental conditions, including nutrient availability, pH, O_2_ and CO_2_ levels, and immune cell presence, due to its flexibility and capacity to rapidly adjust to environmental changes [39]. Additionally, it can specialize in certain micro-niches to optimize the use of available resources [40]. *C. albicans* also enhances its virulence through adherence to biological and inert surfaces [5,40,41,42,43] and the secretion of hydrolases that allow it to exploit host components for growth and nutrition [44,45,46]. Even though *C. albicans* is the most common species in most areas of the world, the frequency of non-albicans diagnoses has been on the rise in recent decades [19,20,47,48], being responsible for more than 50% of cases [49].

### 1.2. C. parapsilosis

*C. parapsilosis*, first isolated in 1928 from the stool of a patient with diarrhea in Puerto Rico [50], is also present in non-human environments such as domestic animals, insects, soil, and marine ecosystems [51]. This diploid yeast has eight chromosome pairs and a genome size of 13.1 Mb, with only 1.83% of its genome characterized so far [52], as its biology has not been as extensively explored as that of the closely related species *C. albicans*. Unlike *C. albicans*, *C. parapsilosis* does not form true hyphae and exists only as yeast or in pseudo-hyphal forms [53] and colonizes the human skin and mucosal membranes as a commensal microorganism [51,54]. The virulence of *C. parapsilosis* is primarily attributed to its ability to adhere to both biotic and abiotic surfaces, a crucial feature for biofilm formation [55,56]. Like other *Candida* species, this yeast produces and secretes several hydrolytic enzymes (lipases (LIPs), secreted aspartyl proteases and phospholipases), which are closely linked to its pathogenic features such as adhesion, cell damage, and tissue invasion [55]. Additionally, its ability to grow in hyperalimentation solutions increases the infection risk posed by this pathogen [57]. In southern Europe, southern America, India and Pakistan, *C. parapsilois* is more common as a cause of candidiasis [58,59] and it has mainly been responsible for the increasing incidences of non-*albicans Candida* infections in the past few years [16]. Infections related to this pathogen are common among neonates with low birth weights, immunocompromised individuals such as HIV and surgical patients (especially those with GI tract surgery), and patients with central venous catheters or other indwelling devices, where *C. parapsilosis* can adhere to [56,60,61]. It accounts for one-third of neonatal *Candida* infections, with a mortality rate of around 10%, and poses a particularly high risk for low-birth-weight neonates [62].

### 1.3. C. auris

Another *Candida* species of increasing interest is *C. auris*. Although this novel and emerging pathogen was first identified in Japan in 2009, according to the European Center for Disease Prevention and Control (ECDC), *C. auris* poses an emerging threat to public health (systems) [63,64]. In 2014, five years after the discovery of the pathogen, *C. auris* bloodstream infections were already reported in South Korea, India and South Africa [65,66,67]. Further instances of rapidly spreading, high-mortality infections have been documented in regions including Europe (the United Kingdom, Spain, Italy), Asia (India, Pakistan), Latin America (Colombia, Venezuela, Panama), and the USA. In 2016, the ECDC requested all local, state and national health departments to report all emerging cases of *C. auris* infections to highlight the emerging threat posed by this pathogen. In September 2017, 127 confirmed and 27 potential cases were reported across 10 states [68,69,70]. In the meantime, the pathogen was isolated in numerous countries, including South Africa, Kuwait, Malaysia, Kenya, Norway, Germany, Oman, Spain, Israel, Venezuela, Brazil, the United States and Canada [65,67,68,71,72,73,74,75,76,77,78,79]. This rapid spread of the fungus is mainly enabled by its ability to persist on human skin and environmental surfaces for weeks, thus causing large outbreaks especially in healthcare facilities through easy skin-to-skin transmission and facilitating inter- and intra-hospital clonal transmission [64,80,81,82,83]. Another alarming finding in the context of *C. auris* infections is the rapid development of (multi-) drug-resistant species, including isolates that show lower sensitivities to all three classes of antifungal drugs [84]. This represents an unprecedented challenge in the treatment of fungal infections [84]. As this pathogen has spread impressively fast since its first discovery to such a considerable extent, the underlying development of mechanisms of resistance is still poorly understood [85]. However, it has recently been shown in studies that azole resistance is linked to clade-specific mutations [86]. Furthermore, the presence of resistance genes on different alleles suggests that the development of *C. auris* resistance is more likely to develop through acquisition than being innate [87]. Morphologically, *C. auris* shows a high similarity to *C. parapsilosis* and other closely related species, which has already led to misidentification of the pathogen, using commercial biochemical diagnostic methods and ultimately to a high rate of failed treatment. In comparison to other *Candida* spp. infections, this resulted in longer ICU stays, underlying respiratory conditions, vascular surgery, prior exposure to antifungal drugs, and lower APACHE II scores in the case of *C. auris* bloodstream infections [88]. Also, *C. auris* shows, in comparison to other *Candida* species, a typical ovoid, ellipsoidal or elongated form and no hyphae [63,65,66,89,90,91,92,93,94]. This fungus, however, is capable of undergoing a tristable phenotypic switch between the regular yeast form, filamentation-competent yeast, and filamentous cells, which is induced by passage through the murine model of systemic candidiasis [92]. Filamentous cells of *C. auris* appear similar to the true hyphae produced by *C. albicans*, but they have distinct biological properties. In terms of metabolism, these cells are more active than yeast cells [92]. Furthermore, this fungus can occur individually or as aggregates. Aggregates are generally more tolerant to antifungal agents, whereas single cells show a higher virulence [95]. Ploidy switching has also been observed under certain conditions. The switch from a haploid to a diploid yeast allows a high degree of adaptation to different environments, as it leads to alterations in various biological characteristics, such as colony size, cellular appearance, color and global gene expression profile. In addition, diploid cells show a higher virulence in mouse models than haploid ones [96]. In contrast to the typical virulence factors of other *Candida* spp., such as phospholipase and proteinase production, germination, adherence, and biofilm formation [97], this pathogen is able to produce phospholipase and proteinase [98,99], but the fungus does not form germ tubes, pseudohyphae or chlamydospores [66,73,95] and has a significantly reduced adherence and biofilm formation [99,100], as this pathogen prefers to grow in so-called “clumps” (i.e., aggregates) [95].

## 2. Biofilm

Biofilms represent one of the most ancient life forms on Earth. The discovery of this microbial lifestyle dates to the late 19th century, when scientists like Antonie van Leeuwenhoek first observed microorganisms in dental plaque. However, the significance of biofilms was not fully appreciated until the 1970s when research started to reveal their prevalence and importance in various environments, including natural aquatic systems, industrial processes, and medical settings [101]. The understanding of biofilms evolved through advances in microscopy and molecular techniques. Early studies focused on the physical and chemical properties of biofilms [102], while later research highlighted their ecological roles and implications for human health [103,104,105,106]. Around 80% of all microorganisms are known to attach to biotic or abiotic surfaces and form a sessile community. This term describes an organized structure of microbial cells enclosed in an extracellular matrix (ECM) [107]. Living in a community like this can protect the pathogenic cells from the host’s immune defense and antibacterial and antifungal drugs and provides a certain degree of stability in a self-controlled microenvironment [108]. These biofilms can consist of either a single species or can host a mixed culture of bacteria and yeasts [109] with poorly understood microbial interactions between the different biofilm members. The coexistence of different organisms within a biofilm complicates the treatment of such infections, as antimicrobials are often specifically directed against one species, non-target organisms still thrive, and infection continues during treatment [110]. Biofilms composed of mixed cultures, including *Candida* spp. and pathogenic bacteria such as *Pseudomonas aeruginosa* and *Staphylococcus aureus,* have been found on implanted medical devices like urinary bladder catheters and central venous catheters [111].

Methods to analyze microbial biofilms at different complexity levels (see Table 1) have advanced significantly over the years and are used for both bacterial and yeast biofilms. Traditional approaches include culture-based techniques, which often underestimate biofilm presence due to the difficulty of culturing microorganisms in a biofilm state [112]. More recent methods utilize molecular techniques, such as polymerase chain reaction (PCR) and next-generation sequencing, to quantitatively analyze biofilm communities [113,114]. These methods enable researchers to identify the diversity and composition of microbial populations within biofilms, providing insights into their ecological dynamics. Additionally, imaging techniques like confocal laser scanning microscopy (CLSM) allow for the visualization of biofilm architecture and composition in situ, providing insights into their complex structures [115,116,117]. Other advanced imaging techniques, such as scanning electron microscopy (SEM) and atomic force microscopy (AFM), further elucidate the physical characteristics and interactions within biofilms [118,119]. These tools have been crucial for understanding biofilm morphology, thickness, and the spatial organization of different microbial species [120].

An important question is the feasibility of analysis methods in clinical settings, i.e., which degree of efficiency a technique can reach with respect to required personnel and equipment for the diagnosis and analysis of biofilms ideally at the bedside of patients. Taking this into account, two groups of methods originating from different eras of diagnostic research appear to be mentioned. Traditional plate count methods (on selective media) offer an undoubted high level of applicability in every microbiological laboratory in clinics but may underrepresent biofilm load in certain clinical samples with low to moderate expected sensitivities and specificities [121]. In contrast, qPCR requires specific (and economically expensive) equipment and personnel with more specific training, but this quantification method can be regarded as highly sensitive for detecting *Candida* biofilms and monitoring treatment effects combined with a high specificity, especially when biofilm-relevant genes are measured, qualifying it as the method of choice for quantification and monitoring in both research and clinical use [122,123,124]. In light of this, it appears clear that there is an urgent need to implement or develop novel reliable and precise diagnostic tools capable of detecting biofilms at the bedside; unfortunately, until now, this has been challenging as most methods are demanding, highly specialized and technically complex.

**Table 1:** Detection methods for microbial biofilms (nicely reviewed in [125]).

Type	Method	Description	Reference
Cell staining assays	Crystal violet (CV) assay	CV binds to negatively charged molecules. After staining, the adsorbed CV is eluted using a solvent. The amount of dye solubilized by the solvent (measured by optical absorbance at 590 nm) is directly proportional to biofilm size.	[126,127]
	1,9-dimethyl methylene blue (DMMB) assay	DMMB binds to the biofilm EPS, which is the intercellular polysaccharide adhesin (PIA), composed of poly-b-1,6-linked-N-acetylglucosamine. After complexation of DMMB with polysaccharides of biofilm, the addition of a decomplexation solution enables the quantification of the released amount of DMMB dye spectrophotometrically.	[128]
	Fluorescein-di-acetate (FDA) assay	After uptake into the cell, FDA is hydrolyzed by cellular esterizes to fluorescein, which can be measured spectrophotometrically.	[129,130]
	LIVE/DEAD BacLight assay	This assay to discriminate live from dead cells is based on the use of two different nucleic acid binding stains. The first dye is the green-fluorescent Syto9, able to cross membranes and bind to DNA. Propidium iodide, the second dye, is red-fluorescent and penetrates only damaged bacterial membranes. The stained samples are analyzed by fluorescent optical microscopy to distinguish between live and dead bacterial populations.	[131,132]
	Resazurin assay	Resazurin (7-hydroxy-3H-phenoxazin-3-one-10-oxide) is a blue, non-fluorescent dye that is reduced by cellular metabolic processes into pink-fluorescent resorufin. The fluorescence of resorufin can be measured spectrophotometrically. These characteristics make resazurin a valuable tool for detecting viable microorganisms and determining the number of viable cells in biofilms.	[133,134,135]
	XTT assay	Using a redox indicator, the XTT method allows for spectrophotometric enumeration of viable cells in biofilms.	[129]
	BioTimer assay (BTA)	Colorimetric assay allowing counting of viable bacteria or yeasts in biofilms. The BTA contains phenol red. Microbial products of primary fermentative metabolism cause a color change from red to yellow. The time required for color switch correlates to initial bacterial or yeast concentration.	[136]
Genetic assays	PCR; qRT-PCR	This PCR-based method allows identification of specific genetic sequences related to individual species. One of the most sensitive and powerful gene analysis methods today is “Real-Time Quantitative Reverse-Transcription PCR” (qRT-PCR). In this method, the fluorescent signal is measured in real time at each amplification cycle and is directly proportional to the amount of amplicons generated.	[137]
	FISH	Fluorescence in situ Hybridization (FISH) is a genetic technique that utilizes oligonucleotide probes tagged with fluorescent dyes. These probes can be specifically designed to bind rRNA, a specific molecule that indicates a target of interest.	[138]
Physical assays	MS; DESI	Mass spectrometry (MS) is a technique used to quantify known substances and to determine the chemical properties of various molecules. In this process, the substance is exposed to an electron beam, ionizing the molecules and producing gaseous ions. These ions are then separated in the mass spectrometer and identified based on their mass-to-charge ratios and relative abundances. The resulting data provide a mass spectrum that is characteristic of each compound and directly reflects its chemical structure. MS offers both qualitative and quantitative capabilities, making it useful for identifying and quantifying unknown compounds. The Desorption Electro-Spray Ionization (DESI) assay enables direct, non-destructive analysis of complex samples, facilitating the chemical characterization of microbial biofilms in various growth states and conditions.	[139]
	CLSM	Using confocal laser scanning microscopy (CLSM) technology, thick biological samples, such as microbial biofilms, can be scanned by capturing images in a line-by-line fashion along the X, Y, and Z axes.	[140,141,142]
	CRM (Confocal RAMAN Microscopy)	The sample is exposed to an electromagnetic laser beam with a known wavelength. By measuring the scattered radiation and energy shifts, the chemical characteristics of the sample can be identified. This method facilitates the capture of the chemical fingerprints of different biofilms.	[143,144,145]
	EM	Electron microscopy (EM) methods exploit the high resolution provided by electron beams, which utilize short-wavelength, high-energy radiation. Transmission electron microscopy (TEM) is particularly effective for imaging the interior of biofilms and their intracellular components. Scanning electron microscopy (SEM) is widely used to visualize the surfaces of microcolonies and mature biofilms. Coupling SEM with focused ion beam (FIB) technology allows for the examination of biofilm interiors by removing surface layers or cutting cross-sections. Both SEM-FIB and TEM can be complemented with energy-dispersive X-ray spectroscopy (EDX), which enables the acquisition of local compositional spectra and maps of bacterial cells and biofilms.	[146,147]
	XM	In X-ray microscopy (XM), the sample is exposed to soft X-ray radiation, either mono- or poly-chromatic, which is focused for high-resolution imaging and compositional mapping. This technique enables detailed analysis of biological samples with minimal preparation and less radiation damage.	[148]
	SPM	Scanning probe microscopy (SPM) reconstructs topographical details of the sample by analyzing the signal from a sharp, nanometer-scale probe that scans the sample near its surface.	[149,150,151,152]

In the context of *Candida* spp. infections, biofilms are one of the main virulence-associated physiological traits and a serious threat to the infected individual [47,153,154,155,156,157]. Compared to infections caused by *Candida* strains preferring the planktonic lifestyle, those pathogens living in these sessile communities show at least twice-as-high mortality rates [158,159] and pose a serious threat as a permanent source of infection [160], causing spread of infection into the bloodstream [161] and increased resistance to antifungal therapies [162].

The potential of biofilm formation varies widely between different *Candida* strains. Various studies have been performed to investigate the formation of biofilm among different *Candida* isolates, showing significantly less biofilm formation among less pathogenic *C. parapsilosis* isolates than among isolates belonging to the highly pathogenic species *C. albicans* [163]. However, differences in biofilm formation were also observed between the isolates within the same species, which directly correlated with the pathogenicity of the respective isolate [164]. Furthermore, it has been observed that an increased pathogenicity of *C. albicans* isolates resulted not only from the increased amount of biofilm formed, but also from the heterogenic composition of this complex formed by conventional yeast cells, hyphae and pseudohyphae [165,166].

### 2.1. Biofilm Formation

**Figure 1:**
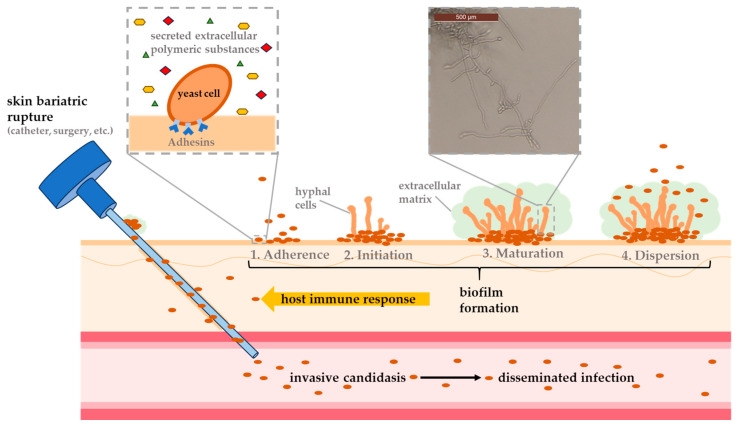
Schematic representation of biofilm development based on the four main steps (adhesion, initiation, maturation and dispersion) and an exemplary illustration showing typical invasive candidiasis infection caused by skin lesions upon the use of, e.g., medical devices.

The formation of a biofilm is a highly regulated process and usually occurs over a period of 38 to 72 h. This process is significantly dependent on the species undergoing its development. In general, however, biofilm formation can be divided into three main phases: First, a planktonic cell adheres to a biotic or abiotic surface, followed by proliferation of the cells and formation of a mature biofilm (Figure 1). The dispersion of cells from the biofilm into the surrounding tissue to colonize new host niches also plays an important role during infection [55].

During the first stage of biofilm formation, lasting 10 to 12 h, planktonic cells adhere to a surface, aggregate into microcolonies, and form a basal monolayer [167,168]. It depends on the species to which surface the cells can attach to the best. *C. albicans*, for example, can adhere much more efficiently to epithelial cells of the GI, urinary tract or blood vessels [169], whereas *C. parapsilosis* tends to form biofilms on abiotic materials like central venous catheters [170,171]. This process is enabled by adhesins (glycoproteins located on the cell wall), which mediate interactions between cells and surfaces [169,172,173]. After the formation of the first layer, present cells undergo species-dependent morphological changes (forming yeast cells, filamentous cells and young hyphae), which can last up to 19 h. During this period, the number of cells increases and macrocolonies are formed. This process is strongly dependent by the species’ capacity to produce extracellular polymeric substances (EPSs), like polysaccharides, lipids and proteins [167]. Approximately 72 h after biofilm formation begins, this process is complete. The result is a complex 3D structure of several layers of polymorphic cells embedded in an ECM formed by exopolimeric material. This network includes numerous pores and water channels, which ensure the smooth circulation of molecules supplying the biofilm components. EPS also plays an important role at this stage. Depending on the carbon source available, these molecules ensure the integrity of the ECM (40% polysaccharide content), which protects the cells from phagocytosis and drug diffusion [174]. During biofilm formation, the detachment of round daughter cells occurs mainly during the maturation phase, resulting in the release of these cells into the surrounding tissue, ready to establish themselves in new host niches [55]. However, such detachment can also occur during the entire formation of a biofilm, leading to the formation of even more robust biofilms than those formed by initial planktonic mother cells [161]. As biofilms mature through successive generations, their virulence potential can grow, presenting a serious threat to both patient treatment and public health [175].

Biofilm development is shaped by mechanisms and factors from both the pathogen and the host, such as the host’s immune status and the yeast’s capacity to interact with the host’s homeostasis [167,176]. In addition, various factors are crucial for the adhesion of the pathogen to a surface of biotic or abiotic nature. These include, for example, the species-specific cell surface hydrophobicity [177], which depend on the glycoproteins on the fibrillar layer (the site of the first contact between the pathogen and the surface) of the cell wall [178,179]. Moreover, the substrate on which the pathogen adheres is another important factor affecting the architecture, morphology and thickness of the biofilm [163]. The interaction of the pathogenic yeast cells with each other, as well as with the substrate, is influenced by physiological conditions such as fluid flow, pH, oxygen concentration and available nutrients [167,180,181]. The fluid flow at the infected site in particular plays a crucial role in nutrient exchange and the integrity of the biofilm by influencing ECM formation [182,183,184,185,186]. Furthermore, the *Candida* species that forms the biofilm and the presence of other microorganisms such as other yeasts or bacteria within the biofilm is important [187]. Several studies using gene disruption, microarray-based transcriptomics, proteomics and genomics have also demonstrated roles of various genes, proteins, DNA and metabolites in the maturation of *Candida* biofilms [188]. These include alcohol dehydrogenase, which controls ethanol acetaldehyde conversion and can thus modulate the biofilm [185].

### 2.2. Biofilm-Specific Resistance Mechanisms

**Figure 2:**
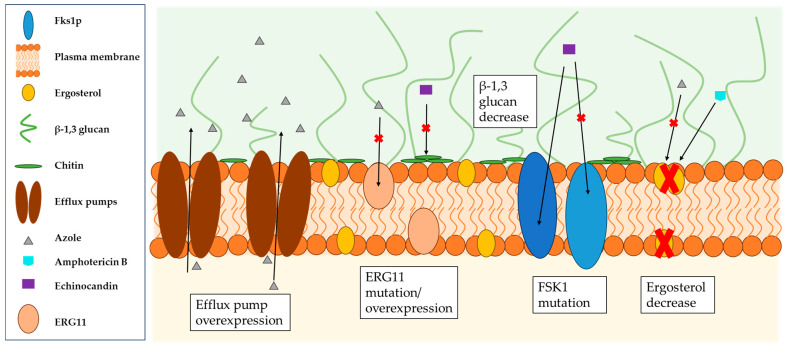
Schematic representation of the biofilm-specific reduced sensitivity of *Candida* species to azoles, amphotericin B and echinocandins due to efflux pump overexpression, ERG11 mutation/overexpression (gene encodes lanosterol 14α-demethylase), the mutation of Fsk1 (gene encodes (1,3)-β-D-glucan synthase) and decreased levels of ergosterol and β-1,3 glycans.

*Candida* spp. infections have been categorized as a high risk to public health due to the high number of resistant planktonic and biofilm-forming isolates against all classes of antifungal therapy causing them [1,189,190,191,192,193,194]. The emergence of such (multi-) resistant pathogens is closely linked to biofilm formation [195,196]. While planktonic cells acquire their resistance by increasing the activity of efflux pumps and mutations in genes encoding the drug target [197,198], biofilm-forming pathogens exhibit additional mechanisms for resistance development which are influenced by the phase of the biofilm within an organism (see Figure 2). For example, planktonic *C. albicans* cells do not show increased sensitivity to antifungal agents, whereas cells of the same isolate forming biofilms show increased tolerance to amphotericin B, nystatin, chlorhexidine and fluconazole [199]. Like planktonic cells, those that form biofilms can overexpress the genes for ATP binding cassette transporters (CDR1 and CDR2) and major facilitator transporter (MDR1) to increase the activity of these efflux pumps and thus prevent the aggregation of azoles (especially fluconazole) in the cells [198,200,201,202,203,204]. Furthermore, biofilm-forming cells have a significantly lower concentration of ergosterol in their cell membrane, especially in the later stages of biofilm formation (cells in mature biofilms contain about half as much ergosterol as planktonic cells) due to an altered transcriptional profile of the sterol pathway to maintain better membrane fluidity [204,205]. However, ergosterol is the target of many azoles and amphotericin B; as such, these cells show a lower sensitivity to those drugs [206]. Moreover, the cell density in the biofilm, but also of cells in general, is supposed to influence antifungal resistance [207,208]. Another key factor contributing to the increased tolerance to antifungal agents is the ECM of the biofilm, shielding the cells from the environment while allowing diffusion of required nutrients, enzymes and water [209,210,211]. Other experiments have shown that biofilms formed under continuous flow have a higher level of ECM than those grown under stagnant or shaking conditions, which is directly associated with increased resistance to amphotericin B, fluconazole and flucytosine [186]. In addition, the ECM has been shown to have the ability to bind or sequester various drugs and thus develop resistance to these [162,212,213,214,215]. Transition to persistent cells is species- and strain-specific, resulting in a change in the cell wall and cell membrane. These cells embedded deep in the biofilm show the highest tolerance to antifungal agents [216,217].

## 3. Detection and Treatment

To initiate a *Candida* infection, these pathogens will face mechanical barriers (e.g., the epithelium), chemical, physical and biochemical antagonists (e.g., pH and antimicrobial peptides), competition among microbes, and the host’s initial and adaptive immune response [218]. As a consequence of their specific cell wall composition, pathogenic *Candida* cells are recognized by the host [219], leading to various immune responses such as the secretion of proinflammatory mediators recruiting specific phagocytes such as polymorphonuclear neutrophils (PMNs), monocytes/macrophages and dendritic cells [220,221,222,223]. However, pathogens of the *Candida* species have developed many preventive mechanisms to avoid recognition by the host, and various adaptations of the pathogen are known to evade clearance by the host immune system [224]. Mononuclear cells in the peripheral blood like macrophages and monocytes have the ability to phagocytize and degrade *C. albicans* cells upon their recognition during infection [225]. However, *Candida* biofilms are resistant to this immune response. In fact, monocytes can become embedded within biofilms, inadvertently strengthening the biofilm structure [226,227]. Additionally, biofilm cells hinder macrophage migration and induce a cytokine response in macrophages that differs from the response triggered by planktonic cells [30]. Interestingly, unlike the neutrophil response, the disruption of *Candida albicans* glycosylation (and consequently ECM disruption) does not affect macrophage migration, suggesting that biofilm cells, rather than the extracellular matrix, are key in influencing macrophage activity [226,227,228]. Moreover, although biofilms trigger elevated levels of proinflammatory cytokines like IL-1β and MCP-1, these do not enhance anti-biofilm activity [229]. Similarly to neutrophils, biofilms also provoke an IL-10 response in macrophages, which exacerbates the issue by promoting biofilm persistence [230]. *C. auris*, which is an example of a non-naturally filamentous yeast, can still form strong multicellular aggregates during infection. These biofilm-like structures show increased resistance to macrophages and host-derived antimicrobial peptides compared to the sensitive planktonic yeast form [231]. Disrupting *C. auris* clumps into single cells reduces this resistance, allowing more efficient macrophage phagocytosis. This emphasizes the fact that large biofilm-associated cell aggregates—regardless of whether they are filamentous—render immune cells less effective at combating biofilm formation [226]. The fact that the organism is already present in small numbers in the host’s healthy gut is an advantage, as various *Candida* spp. are individually adapted to different niches [232,233]. Furthermore, *C. albicans* can reduce phagocytosis by the host’s immune cells through their morphogenesis alone [234]. If a *Candida* cell is recognized and phagocytosed despite these mentioned mechanisms, the environment within the phagosome triggers a stress response [233]. Using their metabolic flexibility, *Candida* spp. can quickly adapt to different available nutrients and switch their metabolism accordingly [233,235,236]. In addition, they have developed effective and extended redundant mechanisms to deal with oxidative, nitrosative and osmotic stress, tightly regulated to respond immediately and strongly to avoid clearance [237]. Taking this into account, it is clear why the US Centers for Disease Control and Prevention classify *Candida* spp. as a serious threat to human health [1].

### 3.1. Detection

A timely diagnosis of *Candida* infections and rapid treatment can be life-critical [238,239]. This includes identifying not only the *Candida* species causing the infection but also the type of infection to determine the mode and length of treatment [240,241,242]. Currently, the gold standard for diagnosing candidiasis is the culture of blood and sterile sites [243], with a sensitivity of approximately 5% depending on the *Candida* species, and pre-treatment of patients with antifungal agents [244]. The major disadvantage of these methods is the time they take to report a positive result, which is 2 to 3 days [238,239,244]. This causes a delay in treatment, as *Candida* has a growth time of 24 h [27]. The varying degree of sensitivity also shows the urgency of other diagnostic methods [242]. Faster, non-culture methods for identifying Candida infections include mannan and anti-mannan antibody detection [30,242,245], β-D-glucan (BDG) detection [230], *C. albicans* germ tube antibody (CAGTA) detection [246], PCR detection of *Candida* DNA [247] and the T2 magnetic resonance (T2MR) *Candida* test [239,240]. These methods also have their limitations; therefore, a combined diagnosis is recommendable [243,248,249,250].

### 3.2. Classic Therapeutic Options

The classic fungicides used for anti-*Candida* treatment include polyenes, azoles, echinocandins and (rarely used) flucytosine [251].

#### 3.2.1. Polyenes

Amphotericin B (AmB) has been the most commonly used polyene for over 55 years [252]. The function of AmB consists in interacting with the ergosterol on the lipid layer of the fungal cell. Binding to ergosterol activates a cascade of events, including the formation of pores in the cell membrane. The resulting increase in membrane permeability causes potassium ions and other intracellular components to escape, ultimately causing cell lysis [253]. The primary limitation of this agent is its low solubility and the high toxicity it presents to host cells, as ergosterol’s structural similarity to cholesterol in mammalian membranes drastically restricts its long-term use [254]. However, promising studies have shown that lipid-based polyenes, such as liposomal amphotericin B (LAmB), exhibit less toxicity towards host cells and are therefore becoming first-line treatment for several types of invasive fungal infections [255]. Furthermore, lower sensitivities to this drug have already been demonstrated in several *Candida* isolates, especially isolates of the species *C. parapsilosis* and *C. auris* [256,257]. The underlying resistance mechanisms are still poorly understood and therefore not as clear as those of echinocandins and azoles.

#### 3.2.2. Echinocandins

Echinocandins represent a newer class of antifungal agents used for treating invasive fungal infections. Compared to polyenes and azoles, echinocandins offer the best clinical outcomes for *Candida* infections, with an efficacy rate of 70 to 75% [258,259,260,261]. The significantly higher survival probability after echinocandin treatment is associated with a high fungicidal activity against most commonly *Candida* species, a low drug–drug interaction, a high safety profile and a lower incidence of acquired resistance compared to the other drug groups [27,262]. This class of drugs, which includes caspofungin, micafungin, and anidulafungin [30,258,263], is known for its high safety profile and potent antifungal activity. This is attributed to their non-competitive inhibition of (1,3)-β-D-glucan synthase, an enzyme crucial for the synthesis of (1,3)-β-D-glucan, an essential fungal cell wall component [264,265]. Despite their narrower antifungal spectrum, echinocandins are highly effective against the most common *Candida* species, including those that have developed resistance to azole medications [201,266]. However, reports of resistant isolates are also increasing against this class of antifungal agents, including *C. parapsilosis* and *C. albicans* isolates, with raised minimum inhibitory concentrations (MICs) against echinocandins in vitro [266,267,268,269,270,271,272]. The reasons for high MICs include an increase in the chitin content of the cell wall [273,274] or mutations in the genes coding for Fks1p, the target of echinocandin treatment [256,272,275].

#### 3.2.3. Azoles

Azoles, the largest class of antifungal drugs in clinical use, are popular for their broad-spectrum efficacy against Candida species, good safety profile, and high bioavailability [275]. As a result, azoles like fluconazole (FLC), voriconazole (VRC), posaconazole (PSC), itraconazole, and isavuconazole are commonly used to treat invasive candidiasis [276]. One of the reasons for the low toxicity of azoles to human somatic cells is their specific mechanism of action, which involves the inhibition of lanosterol 14α-demethylase (encoded by the ERG11 gene), a key enzyme in ergosterol synthesis [277]. As an essential part of the fungal cell membrane, ergosterol’s synthesis inhibition results in the buildup of the toxic 14α-methyl sterol, compromising membrane integrity and the function of membrane-bound proteins. Although the drug class of azoles shows about 15% lower efficacy than echinocandins, as a first-line therapeutic agent, azoles exhibit higher penetration than echinocandins in some forms of deep-seated candidiasis [278]. Furthermore, azoles are inexpensive and can be administered both orally and intravenously, whereas echinocandins are scarce in resource-limited settings and require once-daily intravenous administration. Moreover, azoles are generally better tolerated than echinocandins, which often show strong side effects such as nephrotoxicity, which can be reduced but not completely avoided by lipid-based forms [279,280]. The main challenge in dealing with this antifungal agent is the high incidence of resistant *Candida* species caused by the widespread use of this class of drugs [281]. Azole resistance develops due to a combination of factors, such as mutations in the ERG11 gene (which encodes the target enzyme), point mutations in the ERG3 gene that modify the ergosterol biosynthesis pathway to create less toxic 14α-methyl fecosterol, and the increased expression of multidrug efflux pumps like CDR1, CDR2, and MDR1, which export azoles from the fungal cell [282,283,284].

#### 3.2.4. 5-Flucytosine

5-Flucytosine is carried into the cell by a cytosine permease and metabolized into a toxic version of uridine triphosphate by a cytosine deaminase or converted into an inhibitor of thymidylate synthase, which reduces the availability of nucleotides for DNA synthesis [285,286]. The greatest threat to this antifungal agent is also the increasing number of *Candida* isolates with secondary acquired resistance of up to 8% after monotherapy [287]. The root causes are mutations in the FCY2 gene, which encodes cysteine permease, or in the FCY1 gene, which codes for cysteine deaminase [287]. Currently, flucytosine is only given in combination with AmB to prevent the further development of resistance [30].

### 3.3. Treatment Options and Promising Approaches

Although the healthcare system and ICU care have generally improved in recent decades, as well as new developments in various fungicides and microbial techniques having taken place, the mortality rates associated with invasive candidiasis have not decreased significantly [262]. This is primarily driven by the rising global incidence of multidrug-resistant isolates linked to invasive candidiasis, which results in reduced efficacy of established treatment options [288]. Because of this, the constant development of new therapeutic options, especially against biofilm-forming *Candida* spp., is essential. Due to the mechanical protection offered by the ECM and other factors, *Candida* spp. generally have lower therapeutic sensitivity within biofilms, highlighting the importance of preventing biofilm formation and targeting biofilm cells for the development of novel treatments [289]. Conventional fungicides such as lipid-based formulations of AmB and echinocandins, including caspofungin, also show anti-biofilm effects, but new therapeutic options should be established due to several factors, such as resistance development and increased toxicities, to enable a therapy that is individually customized according to the type of infection, the pathogen and the patient [290]. Several experimental agents are already under investigation as potential new agents against *Candida* spp., but compared to anti-biofilm research against bacteria, that against yeasts is lagging behind (see Table 2). These include substances like chlorhexidine, filastatin, sodium hypochlorite, zosteric acid, gentian violet, EDTA/ethanol catheter lock solutions, and essential oils [291,292,293,294,295,296,297,298,299,300,301,302,303], but also physical methods such as low-level laser [304], photodynamic therapy [305,306,307,308] and antimicrobial coating of catheters [309,310,311,312]. Probiotics are also currently being investigated as a preventive therapy to boost the patient’s immune system, but also as a supportive treatment during a *Candida* infection, as different types of lactobacilli, for example, exert a strong inhibitory effect on *C. albicans* pathogens [313,314]. However, in most cases, an effect against *Candida* biofilms has not been investigated so far.

#### Antimicrobial Peptides

Antimicrobial peptides (AMPs) represent a promising approach for new therapeutic options, not only for biofilm-forming resistant *Candida* strains, but also for pathogens in general (including bacteria, fungi and viruses) [314,315,316,317]. This class of molecules is characterized by its broad antimicrobial spectrum of activity [318,319]. Due to its occurrence in almost all living organisms as a preserved defense mechanism, such as plants, mammals, arthropods and many others, it is an almost unlimited source of potential new therapeutics against various highly dangerous infectious diseases [320]. Furthermore, these molecules are less susceptible to resistance development than conventional therapeutics due to their special mode of action, distributed growth and modulation of the host immune system [321]. Currently, around 50 AMPs are in clinical trials (see Table 3), including up to 20 against Gram-positive bacteria, about 15 against Gram-negative bacteria and only a few against yeasts such as *Candida* spp. These numbers highlighted the urgent need for investigation into antimicrobial peptides against fungi such as *Candida* spp.

Defensins, found in plants, are critical in defending against microbial infections. These small peptides, which are rich in cysteine, are capable of combating many pathogens, including fungi and bacteria [366,367]. Their primary function is to prevent microbial invasion of plant tissues, making them a vital defense mechanism. Research has shown that plant defensins are generally non-toxic to human cells, which makes them attractive candidates for developing new antimicrobial agents [368,369,370,371,372].

In humans, AMPs such as human β-defensins are mainly expressed in epithelial tissues and play a role in diminishing pathogen virulence by either inhibiting growth or modulating the immune response [373,374,375]. Among these, HBD-3 is recognized for its potent antifungal activity against *C. albicans*, while HBD-1 and HBD-2 also display antimicrobial properties, but to a lesser extent. Interestingly, studies have demonstrated that reducing the disulfide bridges in HBD-1 can enhance its effectiveness against *Candida* species [376,377,378,379,380,381].

Insects and arachnids also contribute to the pool of AMPs, with arthropods being one of the largest sources of these peptides. For instance, the 18-amino-acid peptide gomesin, extracted from the tarantula *Acanthoscurria gomesiana*, exhibits a broad spectrum of activity against various pathogens, including fungi and bacteria. Its low toxicity and effectiveness make it a promising candidate for treating infections such as vulvovaginal candidiasis [382,383,384,385,386,387,388,389].

**Figure 3:**
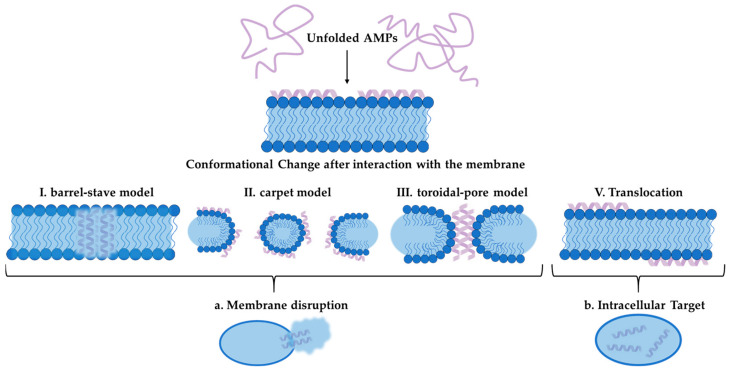
Illustration of AMP mechanism of action. Unfolded AMP interacts with cell membrane and undergoes a conformational change. Afterwards, the peptide can be translocated into the cell to act intracellularly or distribute the membrane following the barrel-stave model, the carped model or the toroidal pore model.

Moreover, AMPs can be isolated from other organisms, such as amphibians and mollusks. The exploration of these peptides is essential, particularly in light of the drawbacks associated with conventional antifungal drugs, which often carry significant side effects and require long-term therapy [390,391,392]. The discovery of new AMPs is imperative, as they may offer effective alternatives with reduced cytotoxicity. Pom-1 and Cm-p5 are two noteworthy AMPs that have garnered attention for their antifungal properties, particularly against *Candida* species [393,394,395]. The peptide Pom-1, isolated from the freshwater snail *Pomacea poeyana*, exhibits a unique α-helical structure when in a membrane-like environment. This peptide demonstrates significant activity against various pathogens, including *Pseudomonas aeruginosa*, *Klebsiella pneumoniae*, and *Listeria monocytogenes*, while also showcasing antifungal activity against *Candida* species [395,396]. Research indicates that while Pom-1 has low activity against planktonic cells of *Candida*, its derivatives, Pom-1A to Pom-1F, display enhanced antifungal effects, particularly against biofilm formation of *C. albicans* [397]. The ability of these derivatives to inhibit biofilm development is particularly promising, as biofilms contribute to the persistence and resistance of fungal infections. The low cytotoxicity of Pom-1 and its derivatives indicates that their mode of action is not based on traditional pore formation (Figure 3). It is assumed that these novel AMPs do not interact with membran lipids, but possibly bind to other membrane epitopes such as membrane proteins, which are essential for biofilm formation. Hence, it has been suggested that, similar to neutralizing antibodies, these peptides simply act by concealing those epitopes from productive interactions of cell–cell or cell–substratum contacts [398]. This model proposes that the peptides bind to specific targets on the pathogenic membrane, disrupting cell–cell interactions and inhibiting biofilm formation, which opens avenues for further research into their precise mechanisms of action [398].

Cm-p5, another compelling AMP, is derived from the coastal tropical mollusk *Cenchritis muricatus* [399]. This peptide has demonstrated specific antifungal activity against *C. auris* and *C. albicans*, including strains resistant to conventional treatments [400,401,402]. The ability of Cm-p5 derivatives to inhibit biofilm formation and affect various *Candida* isolates, including FLC-resistant mutants, makes it a significant candidate in the fight against opportunistic fungal infections. Studies show that Cm-p5 acts by targeting the fungal membranes, leading to alterations that prevent the establishment and growth of biofilms. It has been described that Cm-p5 interacts with *C. albicans* lipid bilayers in a fungistatic mode of action without causing significant perturbation or pore formation, probably fitting the carpet model of action better [400]. This may suggest, although not yet clear in detail, that surface structures required for productive cell–cell or cell–substratum interactions may be sequestered or concealed by the peptide, causing modification of the membrane. This peptide’s effectiveness, combined with its low toxicity, positions it as a promising alternative to existing antifungal therapies [394]. Both Pom-1 and Cm-p5 exemplify the potential of AMPs in combating fungal infections, particularly in an era where antibiotic resistance is a growing concern. Their unique structures and mechanisms of action highlight the importance of exploring natural sources for the development of new, effective antimicrobial agents. As research continues to unveil the intricacies of these peptides, their role in therapeutic applications may significantly impact the management of fungal diseases.

Synergistic effects of AMPs with traditional antibiotics against, e.g., *P. aeruginosa* and other bacterial pathogens, leading to improved antibiotic activity or overcoming bacterial resistance, were described decades ago and have been recognized as a valuable additional option in the treatment of infections, as is nicely reviewed in the paper of Taheri-Araghi [403]. A similar situation has also been described for fungal pathogens like *Candida* species, for which synergistic effects have been observed for AMPs in combination with traditional antifungals like AmB and FLC (nicely reviewed by Mhlongo et al. [404]). Additional recent evidence comes from Kissmann et al. [398], who found that neutralizing peptides lead to reduced biofilm formation and hence enhanced growth in the planktonic phase, rendering the planktonic cells more accessible for traditional AmB and FLC, resulting, in turn, in a “rescuing” of the antifungal agents, and overcoming resistance by even multi-resistant clinical isolates [398].

Overall, the study of AMPs across different species provides valuable insights into their mechanisms of action, therapeutic potential, and the possibility of overcoming antibiotic resistance, making them a focal point for future research in the development of novel antimicrobial therapies. Fascinating new opportunities appear to be opened at the moment by the introduction of artificial intelligence and machine learning approaches for the design of novel (optimized) sequences, likely representing the next generation of highly active molecules [405,406,407].

## 4. Conclusions

In conclusion, the rise in *Candida* infections and their associated morbidity and mortality rates highlight the urgent need for improved diagnostic and therapeutic strategies. The pathogenicity of species like *C. albicans*, *C. parapsilosis*, and *C. auris*, combined with their ability to form resilient biofilms, complicates treatment efforts and underscores the significance of antibiotic resistance. While traditional antifungal therapies have been effective, their limitations and the emergence of resistant strains necessitate the exploration of alternative approaches. AMPs, exemplified by Pom-1 and Cm-p5, offer a promising avenue for combating these infections due to their broad-spectrum activity and unique mechanisms that circumvent traditional resistance pathways. Continued research into the mechanisms of action of these peptides and their potential integration into clinical practice could significantly enhance our ability to manage and treat fungal infections effectively. As the landscape of infectious diseases evolves, the development of innovative therapeutic options will be crucial in safeguarding public health against the growing threat of drug-resistant pathogens.

## Figures and Tables

**Table 2:** Number of publications on the treatment of biofilms of major bacterial and yeast pathogens.

Microbe	Organism	Number of Publications
Bacteria	*Staphylococcus aureus*	~5.000 to 10.000
	*Pseudomonas aeruginosa*	~3.000 to 7.000
	*Escherichia coli*	~2.000 to 5.000
Yeast	*Candida albicans*	~1.500 to 3.000

**Table 3:** Overview of AMPs in clinical trials (nicely reviewed in [322,323]).

AMP	Molecule Type	Target	Mechanism	References
Nisin	Polycyclic lantibiotic	Gram-negative and Gram-positive bacteria	Depolarization of cell membrane	[324]
Gramicidin	Polycyclic peptide	Gram-positive bacteria	Membrane disruption/immunomodulation	[325]
Polymyxin B	Cyclic polypeptide	Gram-negative bacteria	Membrane disruption/immunomodulation	[326]
Polymyxin E	Cyclic polypeptide	*Acinetobacter baumannii*	Membrane disruption/immunomodulation	[326]
Daptomycin	Lipopeptide	Gram-positive bacteria	Membrane disruption/immunomodulation	[327]
LL-37	Human cathelicidin	Gram-negative and Gram-positive bacteria	Membrane disruption/immunomodulation	[328]
Melittin	α-helical peptide	Bacteria, viruses, fungi, parasites, cancer cells	Membrane disruption/immunomodulation	[329]
Friulimicin	Cyclic lipopeptide	Gram-positive bacteria	Membrane disruption	[330]
Murepavadin	Analog of Protegrin	*Pseudomonas aeruginosa*, *Klebsiella pneumoniae*	Binding to LptD	[331]
rBPI21	Cyclic lipopeptide	Gram-negative bacteria	Membrane disruption	[332]
Iseganan	Analog of Protegrin	*Pseudomonas aeruginosa*	Membrane disruption	[333]
Surotomycin	Cyclic lipopeptide	*Clostridioides difficile*	Membrane disruption	[334]
Pexiganan	Analog of Magainin	Gram-negative and Gram-positive bacteria	Membrane disruption/immunomodulation	[335]
XOMA-629	Derivative of BPI (bactericidal permeability increasing protein)	*Propionibacterium acnes*, *Staphylococcus aureus*, *Streptococcus pyogenesand*	Immunomodulation	[336]
Omiganan	Derivative of Indolicidin	Gram-negative and Gram-positive bacteria; fungi	Membrane disruption/immunomodulation	[337]
NVB-302	Lantibiotic	*Clostridioides difficile*	Inhibition of cell wall synthesis	[338]
OP-145	Derivative of LL-37	*Staphylococcus aureus*	Membrane disruption/immunomodulation	[339]
P113	Fragment of Histatin-5	*Plasmodium falciparum*	Membrane disruption/immunomodulation	[340]
LTX-109	Synthetic tripeptide	*Staphylococcus aureus*	Membrane disruption	[341]
EA-230	Oligopeptide	Anti-inflammatory effects to renal ischemia/reperfusion injury (IRI)	Immunomodulation	[342]
SGX942	Analog of IDR-1 (innate defende ragulator)	Oral mucositis in patients with head and neck cancer after cancertreatment	Immunomodulation	[343]
hLF1-11	Fragment of human lactoferrin	Bacterial/fungal infections	Membrane disruption/immunomodulation	[344]
C16G2	Synthetic peptide	*Streptococcus mutans*	Membrane disruption	[345]
Novexatin	Cyclic cationic peptide	Fungal nail infection	Membrane disruption	[346]
Ramoplanin	Glycolipodepsipeptide	*Clostridioides difficile*	Inhibition if cell wall synthesis	[347]
p2TA	Synthetic peptide	Gram-negative and Gram-positive bacteria	Immunomodulation	[348]
D2A21	Synthetic peptide	*Pseudomonas aeruginosa*	Membrane disruption	[349]
Melimine	Chimeric peptide	*Staphylococcus aureus*	Membrane disruption	[350]
Mel4	Derivative of melimine	Gram-positive bacteria	Membrane disruption	[351]
LFF571	Semisynthetic thiopeptide	*Clostridioides difficile*	Inhibition of protein synthesis	[352]
Delmitide	Derivative of HLA (human leucocyte antigen)	Crohn’s disease and ulcerative colitis	Immunomodulation	[353]
DPK-060	Derivative of Kininogen	Gram-negative and Gram-positive bacteria	Membrane disruption/immunomodulation	[354]
GSK1322322	Synthetic hydrazide	*Staphylococcus aureus*	Peptide deformylase inhibition	[355]
PXL01	Analog of Lactoferrin	Postsurgical adhesions	Immunomodulation	[356]
AP-214	Derivative of α-MSH (Melanocyte-stimulating hormone)	Acute kidney injury	Membrane disruption/immunomodulation	[357]
PMX-30063	Defensin mimetic	*Streptococcus pneumonia*, *Streptococcus viridans*	Membrane disruption/immunomodulation	[358]
XF-73	Derivative of porphyrin	*Staphylocoocal* infection	Membrane disruption	[359]
CZEN-002	Derivative of α-MSH	Antifungal	Immunomodulation	[360]
Ghrelin	Endogenous peptide	Chronic respiratory infection	Immunomodulation	[361]
Wap-8294A2	Cyclic peptide	*Staphylococcus aureus*	Membrane disruption	[362]
PL-5	Synthetic peptide	*Escherichia coli*, *Pseudomonas aeruginosa*, *Klebsiella pneumoniae*, *Staphylococcus aureus*, *Staphylococcus epidermidis*	Membrane disruption	[363]
IDR-1	Synthetic peptide	Gram-negative and Gram-positive bacteria	Immunomodulation	[364]
PXL01	Peptide derived from human lactoferrin	Postoperative adhesions	Immunomodulation	[365]

## Data Availability

No new data were created or analyzed in this study.
